# The association of BRAF V600E gene mutation with proliferative activity and histopathological characteristics of congenital melanocytic nevi in children^☆^

**DOI:** 10.1016/j.abd.2022.01.016

**Published:** 2023-05-06

**Authors:** Jianyou Chen, Gaolei Zhang, Xiaoyan Liu, Ping Tu

**Affiliations:** a Department of Dermatology and Venereology, Peking University First Hospital, Beijing, China; b Department of Dermatology, Children’s Hospital Affiliated to Capital Institute of Pediatrics, Beijing, China

**Keywords:** Genes, Immunohistochemistry, Ki-67 Aantigen, Nevus, Pigmented, Proto-oncogene proteins BRAF

## Abstract

**Background:**

A lot of congenital melanocytic nevi (CMN) carry the somatic mutation in the oncogene BRAF V600E. But the detailed histopathologic characteristics and the proliferative activity of CMN with BRAF V600E gene mutation have not been systematically documented.

**Objective:**

To identify the proliferative activity and histopathological features correlating them with BRAF V600E gene mutation status in CMN.

**Methods:**

CMN were retrospectively identified from the laboratory reporting system. Mutations were determined by Sanger sequencing. The CMN were divided into a mutant group and control group according to whether there was BRAF gene mutation and were strictly matched according to gender, age, nevus size, and location. Histopathological analysis, analysis of Ki67 expression by immunohistochemistry and laser confocal fluorescence microscopy were performed.

**Results:**

The differences in Ki67 index, the depth of nevus cell involvement and the number of nevus cell nests between the mutant group and the control group was statistically significant, with p-values of 0.041, 0.002 and 0.007, respectively. Compared with BRAF V600E negative nevi, BRAF V600E positive nevi often exhibited predominantly nested intraepidermal melanocytes, and larger junctional nests, but the difference in this data sets were not statistically significant. The number of nests (p = 0.001) was positively correlated with the proportion of Ki67 positive cells.

**Study limitations:**

A small sample of patients were included and there was no follow-up.

**Conclusions:**

BRAF V600E gene mutations were associated with high proliferative activity and distinct histopathological features in congenital melanocytic nevi.

## Introduction

Congenital Melanocytic Nevi (CMN) defined by their presence at birth or in the first few weeks of life originate from benign melanocytic proliferations of the neural crest. The clinical features of CMN are various such as body site, a more diverse spectrum of colors, and surface topographical changes. Potential complications from CMN include melanoma development and central nervous system involvement termed Neurocutaneous Melanosis (NCM).

The classification of CMN has historically been defined by size alone. Recent recommendations were published based on expert consensus, utilizing Predicted Adult Size (PAS) categories, the number of accompanying CMN, anatomic location, and several topographic descriptors. A small CMN is defined as less than 1.5 cm PAS and medium up to 20 cm PAS (M1: 1.5–10 cm, M2: >10–20 cm). Large and giant CMN are classified as those CMN with PAS between 20 and 40 cm (L1: >20–30 cm, L2: >30–40 cm) and greater than 40 cm (G1: >40–60 cm, G2: >60 cm), respectively.^1^

Small and medium CMN are reported approximately in 1 in 100 births.^2^ while Large and giant CMN has an estimated prevalence of 1 in 20,000 to 1 in 50,00 births and 1 in 200,000 to 1 in 500,000 births respectively.^3^, ^4^, ^5^

CMN harbored predominantly a post-zygotic somatic NRAS or BRAF mutation. Nevertheless, different mutations usually depend on nevus size. In the vast majority of cases, small and medium CMN is more frequently noted to have a BRAF V600E mutation, while large/giant counterparts more frequently harbor NRAS (Q61) mutations.^6^, ^7^, ^8^ CMN are usually benign melanocytic neoplasms, but sometimes it is hard to differentiate CMN from melanoma, especially at an early stage of melanoma. Therefore, biopsies or surgery are needed to make a further pathologic examination. The BRAF V600E mutation is the most common genetic driver in melanoma and encodes a serine/threonine kinase in the RAS/Mitogen-Activated Protein Kinase Pathway (MAPK).^9^, ^10^, ^11^, ^12^

Remarkably, more than 80% of melanocytic nevi harbor BRAF mutations,^10^, ^13^ including the melanocytic nevi that act as precursors for melanoma.^14^

In melanoma, the presence of the BRAF V600E mutation correlates with certain histopathologic features, including intraepidermal upward scatter of melanocytes, nest formation of intraepidermal melanocytes, thickening of the epidermis, and larger tumor cells.^15^ Similarly, melanocytic nevi with a BRAF V600E mutation are associated with a globular dermoscopic pattern and a predominantly dermal histologic growth pattern.^13^, ^16^ These results suggest that the BRAF V600E mutation may affect the characteristics and behavior of nevi.

Mutations in BRAF (v-raf murine sarcoma viral oncogene homologue B) have been detected with high frequency in CMN, especially in small and medium CMN.^17^ However, as far as we know, there are no relevant reports on the effect of BRAF V600E gene mutation in CMN on proliferation activity and histopathology. As such, the study of the biology of CMN may further inform the understanding of pathways leading to melanoma.

In this study, the primary objective was to determine the proliferation activity (by Ki67) and histopathological pattern in CMN with BRAF V600E gene mutation.

## Materials and methods

This study was approved by the institutional review board of the Capital Institute of Pediatrics (SHERLL2020054) and was carried out in accordance with the Declaration of Helsinki.

### Study design and patients

A total of 84 CMN children were recruited from the present dermatology clinics between May 2016 and October 2020. Nevi that were present at birth or within 1 month of birth were included. Exclusion criteria included a diagnosis of blue nevus, spitz nevus, nevus present after 1 month of birth or with an uncertain onset time. Before participating in the study, informed consent was given by the parents.

All 84 CMN were completely surgically removed and bisected, one half of the tissue was formalin-fixed and histopathological diagnosed by a board-certified pathologist (P.X) and assessed according to a method adapted from Marchetti et al.^17^ The second half of the 84 tissues were flash frozen at −80 °C. DNA extraction was performed as previously described^8^ and using the QIAamp DNA Mini Kit (QIAGEN, Hilden, Germany) (n = 24) according to the manufacturer’s protocol. The authors carried out gene mutation tests including BRAF and NRAS.

All the 84 CMN recruited were genetically tested, 12 of which were found to have the BRAFV600E mutation, while the others were negative. Twelve children without BRAFV600E mutation were randomly selected as the control group, and their age, sex, nevus size, and anatomical location were matched with the study group.

All archival tissue blocks had been paraffin-embedded. Hematoxylin-eosin-stained sections from each block were reviewed to confirm the diagnosis.

### BRAF Exon 15 and NRAS sequencing

Tissue samples were treated using the QuickExtract™ FFPE DNA Extraction Kit using standard protocols. Primers used for PCR-amplification of the BRAF Exon 15 were:

Forward: 5′-TCATAATGCTTGCTCTGATAGGA-3′.

Reverse: 5′-GGCCAAAAATTTAATCAGTGGA-3′.

Or forward (shorter amplicon): 5′-TGTTTTCCTTTACTTACTACACCTC-3′.

Reverse: 5′-TAATCAGTGGAAAAATAGCCTC-3′.

PCR-Primers for NRAS Exon 1 were:

Forward: 5′- CGC CAA TTA ACC CTG ATT ACT-3′.

Reverse: 5′- CAC TGG GCC TCA CCT CTA-3′.

PCR-Primers for NRAS Exon 2 were:

Forward: 5′-GATTCTTACAGAAAACAAGTG-3′.

Reverse: 5′-ATGACTTGCTATTATTGATGG-3′.

Successful amplification of the respective region was confirmed by visualizing 5 µl of the PCR products on a 2% Agarose Gel containing GelRed™ Nucleic Acid Gel Stain. PCR products were cleaned up using USB® ExoSAP-IT® PCR Product Cleanup (Affymetrix®, Santa Clara CA), and Sanger-sequencing was performed using the respective primers used in PCR.^18^

### Histopathological analysis

Hematoxylin-eosin-stained slides and tissue blocks were available for 24 cases. The hematoxylin-eosin-stained slides were reviewed by 2 board-certified dermatopathologists (P. T and G.L.Z) before the availability of the Ki67 staining results. Histopathological analysis without knowledge of the gene mutation result was also carried out by the 2 board-certified dermatopathologists (P. T and G.L.Z) on hematoxylin and eosin-stained sections to quantify the following parameters: 1) Histopathological classification (junctional, compound or intradermal), 2) Predominant microanatomical growth pattern, 3) Nest formation of junctional melanocytes; 4) The depth of nevus cell involvement: ① Within the epidermis, ② In the superficial dermis, ③ In the middle dermis, ④ In the deep dermis, ⑤ In the subcutaneous tissue; 5) Architectural characteristic and cytological atypia. The microanatomical growth pattern was quantified as: (a) Predominantly epidermal, (b) Epidermal and dermal components almost equivalent, and (c) Predominantly dermal. The presence of any architectural disorder and/or cytological atypia was rated as absent or present.

### Immunohistochemical evaluation of proliferative activity of the nevus cells

Paraffin blocks of 24 lesions of CMN were retrieved. Immunohistochemical detection of the Ki67 was performed. The antibody was a mouse monoclonal antibody (Clone OTI8H5, ZSBG-Bio, CHINA) against humans. The number of Ki67-positive nevus cells was counted at 400× magnification field (The actual surface area covered per field was 0.11 mm^2^). Ki67-positive nevus cells could be differentiated from epidermal keratinocytes because of nest formation by the nevus cells at the dermo-epidermal junction. Ten fields were randomly selected to record the number of positive nevus cells. The average number of positive cells indicated the positive rate, and the location and distribution pattern of positive cells was evaluated.

IHC studies were carried out on 5 µm thick sections of formalin-fixed, paraffin-embedded tissue. All sections were stained for Ki67 protein by immunohistochemical pv-9000 two-step method under the same condition. The sections were routinely dewaxed and hydrated, repaired under high pressure, endogenous peroxidase blocker was added. Incubated at room temperature for 10 min, wash with distilled water, soak, and wash with PBS 5 min for 3 times. Added Ki67 primary antibody (Clone OTI8H5, ZSBG-Bio, CHINA, 1:100 dilution) and incubate at 37 °C for 1 h. Soak and wash with PBS for 5 min, twice paraffin sections with appropriate positive and negative controls. Presence or absence of Ki67 staining was identified with 100% consensus agreement (P. T and G.L.Z).

### Laser confocal fluorescence microscopy (LCFM)

**Figure 1:**
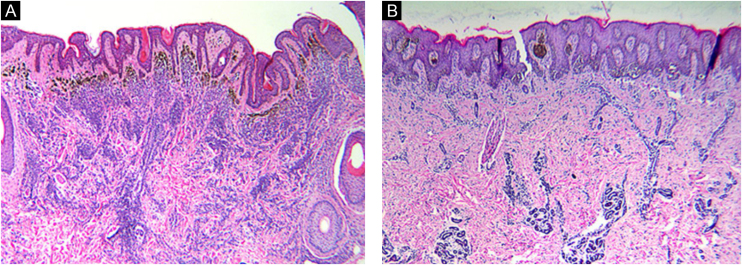
Histomorphologic features of CMN. Congenital growth pattern in nevi involving adnexal epithelium and wrapping of nevus cells around vessels and splaying of melanocytes in-between collagen bundles (A‒B). But the nevi with BRAF V600E mutation (A) would spread into deep dermis even deeper than wild type nevi (B). (A‒B, Hematoxylin & eosin, ×40).

If there were too many melanin particles in the specimen, which affected the judgment of the results, the laser confocal fluorescence microscope was used to further clarify the diagnosis. Sections were deparaffinized in xylene and rehydrated with 100%, 95%, 85% and 75% ethanol for 5 min respectively and rinsed with phosphate-buffered saline for 5 min for 3 times. After microwave heat-induced epitope retrieval for 15 min and blocking in 5% normal goat serum for 1 hour at room temperature, sections were stained with monoclonal mouse anti-human Melanin A and rabbit antihuman Ki-67 antibody (Abcam, Cambridge, MA, USA) in 5% bovine serum albumin/Tris-buffered saline. For detection, AlexaFluor 488-labeled goat anti-mouse and AlexaFluor 595-labeled goat anti-Rabbit antibodies (Abcam, Cambridge, MA, USA) and DAPI were used. For colocalization analysis, cells were simultaneously labeled with anti-Melanin A mouse antibodies and anti-Ki-67 rabbit antibodies. and detected with AlexaFluor 594-labeled goat anti-mouse or AlexaFluor 488-labeled goat anti-rabbit antibodies. The procedure was performed manually. Positive and negative controls were included with each run of samples. The percentage of positive cells was counted under a high-power view (Fig. 1).

### Data analysis

Statistical analysis was performed using SPSS v. 22. Descriptive statistics were stratified by BRAFV600E gene mutation status, with mean and Standard Deviation (SD) for continuous variables. The paired *t*-test was used to compare the means of continuous data. The significance tests of correlation and partial correlation coefficients between a binary histopathological feature and the Ki67 index were performed. All given p-values are two-tailed and a p-value < 0.05 was considered statistically significant.

## Results

Data on the 12 matched pairs including patients (n = 24) and nevi (n = 24) are shown in Table 1. Characteristics of CMN of mutation group and controls. The mutant and control groups were closely matched in age, sex, and location. There were no statistically significant differences in age, sex, history of melanoma, and history of non-melanoma skin cancer between the mutant and control groups.

**Table 1:** Characteristics of nevi in mutant and control groups.

Nevi pairs^a^	Sex	Age (yr)	Anatomic site	Nevus size (cm)	PAS	Ki67	Deep level	Size of junctional nests (µm)	Number of junctional nests	Histopathologic subtype	Architectural disorder or cytological atypia
1a	F	3	Leg	2.2	M1	3	3	1056	10	Compound	Absent
1b	F	5	Leg	4	M1	3	2	339	3	Compound	Absent
2a	F	1	Back	3.5	M1	10	2	895	20	Compound	Absent
2b	F	1	Back	2.5	M1	5	2	801	6	Compound	Absent
3a	M	0.3	Abdomen	3	M1	10	3	915	21	Compound	Absent
3b	M	0.5	Abdomen	4.5	M1	1	1	1686	11	Compound	Present
4a	F	0.3	Chest	2.4	M1	3	2	1192	20	Compound	Absent
4b	F	0.3	Chest	2	M1	4	1	781	13	Compound	Absent
5a	M	7	Leg	4	M1	3	3	406	5	Compound	Absent
5b	M	5	Leg	5.2	M1	2	2	619	3	Intradermal	Absent
6a	M	6	Waist	1.7	M1	1	3	570	8	Compound	Absent
6b	M	8	Waist	2.5	M1	1	2	484	3	Compound	Absent
7a	M	0.6	Leg	4	M1	1	2	2900	8	Compound	Absent
7b	M	0.8	Leg	2.4	M1	1	1	393	3	Compound	Absent
8a	F	10	Leg	1	M1	3	2	547	2	Intradermal	Present
8b	F	9	Leg	1.3	M1	2	2	770	1	Intradermal	Absent
9a	M	0.3	Forearm	3.8	M1	3	3	1468	20	Compound	Absent
9b	M	0.4	Forearm	2	M1	0	3	417	2	Compound	Absent
10a	F	4	Trunk	2.6	M1	6	2	1744	7	Compound	Absent
10b	F	3	Trunk	6	M1	10	2	660	12	Compound	Present
11a	F	3	Trunk	5.5	M1	4	3	1103	18	Compound	Present
11b	F	3	Trunk	3	M1	2	2	681	8	Compound	Absent
12a	M	1	Leg	4.3	M1	2	3	867	7	Compound	Absent
12b	M	1	Leg	3.7	M1	1	1	448	8	Compound	Absent

a For nevi pairs, a = cases with BRAF V600E mutation; b = control; F= Female; M= Male; PAS= Predicted Adult Size; M1= 1.5 - 10 cm; Deep level: 1-Within the epidermis, 2-In the superficial dermis, 3-In the middle dermis, ④ In the deep dermis.

There were 21 compound nevi in this study (21 of 24, 87.5%). Nevertheless, there was only one intradermal nevus in the mutation group (8.3%), while two were in the control group (16.6%).

**Figure 2:**
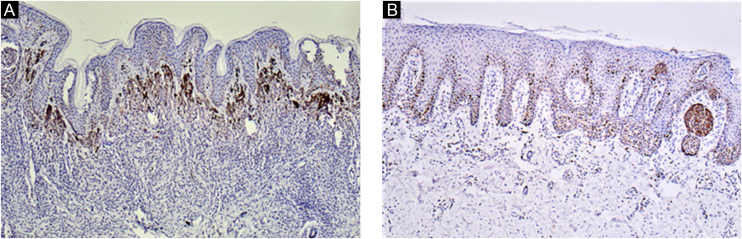
Ki67 immunohistochemistry of CMN. Ki67 staining was restricted to the nucleus of positive cells in a distinct granular pattern of staining and more frequently observed at the dermal-epidermal junction (A‒B, ×100).

**Figure 3:**
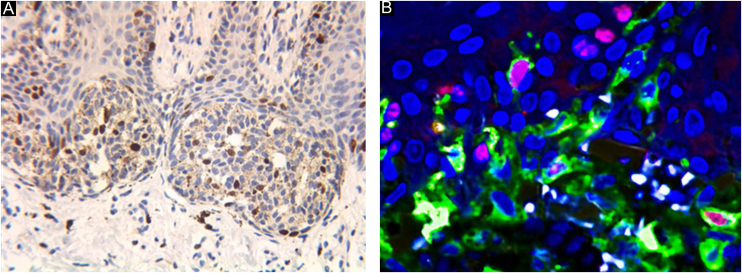
The brown stained nuclei indicate Ki67 positive expression (A, ×400). Confocal laser fluorescence microscopy can easily eliminate the interference of melanin granules. Cells with red fluorescence in the center and yellow fluorescence around are Ki67 positive cells (B ×600).

Ki67 staining was restricted to the nucleus of positive cells in a distinct granular pattern of staining and was often inhomogeneous. Occasionally the nucleolar region was stained prominently. No cytoplasmic staining was observed. More frequent staining was observed at the dermal-epidermal junction (Figure 2, Figure 3).

Table 2 shows the proportion of cells positive for the Ki67 staining in each nevi pair. Statistically significant differences were detected between the mutation group and the control (p = 0.041).

**Table 2:** Statistical characteristics of nevi in mutant and control groups.

	Cases with BRAF V600E mutation		Control		p-value
	Mean	SD	Mean	SD	
Depth level	2.58	0.51	1.75	0.62	0.002
The number of nests	12.16	7.03	6.08	4.20	0.007
The size of nests	1138.58	672.95	673.25	357.84	0.080
Ki67	4.08	3.06	2.25	1.65	0.041

SD, Standard Deviation.

The differences in the depth of nevus cell involvement and the number of nevus cell nests between the mutant group and the control group were statistically significant, with p-values of 0.002 and 0.007, respectively, while the difference in the size of cell nests between the two groups was not statistically significant (Table 2).

**Figure 4:**
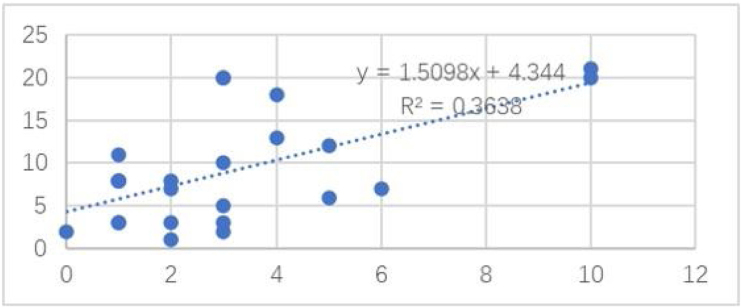
Correlation between Ki-67 and nest numbers.

Among the cases, the correlation between the number of the nevus cell nests and the Ki67 positivity rate is shown in Fig. 4. The number of the nevus cell nests (Linear regression analysis, Spearman’s correlation coefficients: coefficient = 0.603; p = 0.001; Fig. 1) was strongly correlated with the proportion of cells positive for Ki-67 staining. However, Ki-67 staining had no significant correlation with nevus cell depth, nevus cell nest size, and nevus size, and the correlation coefficients were 0.11, 0.15 and 0.01, respectively.

BRAF immunohistochemical staining was performed in 10 cases in a blinded manner (i.e., without knowledge of the results of the gene sequencing before analysis of the immunohistochemical staining data). All 7 nevi with negative immunohistochemical staining for BRAF V600E were negative for BRAF mutation, and all 3 nevi with positive immunohistochemical staining for BRAF V600E had BRAF V600E gene mutation. No other recurrent somatic mutations were identified.

## Discussion

At present, there are only a few literature reports about the association of gene mutation with the pathology and proliferation activity of CMN.^13^, ^19^, ^20^ These reports were holistic studies and did not distinguish between congenital and acquired nevi. To our knowledge, this is the first study to demonstrate the relationship between BRAF mutation on the proliferation activity and histopathology of CMN.

According to microscopic histologic characteristics, CMN may be junctional, compound or exclusively intradermal. The histologic characteristics are related primarily to the size of the nevus.^21^ Nearly all nevi included in this study were compound (21 of 24, 87.5%) and the histopathological features were consistent with the congenital pattern, such as symmetry, good circumscription, uniform nests in the epidermis equidistant from each other, nests of melanocytes and individual melanocytes in the dermis that mature with their descent, wrapping of nevus cells around vessels and adnexal structures, and splaying of melanocytes in-between collagen bundles.^22^ It's worth noting that the difference in nevus depth was significant between the two pair-matched groups. The nevus cells in the BRAF mutation group extend deeper than the non-mutation group. This phenomenon may be caused by the enhanced proliferative activity of nevus cells after the BRAF gene mutation. The depth of nevus infiltration was also positively correlated with the size of the nevus.^21^ By reason of the foregoing, the authors can draw a preliminary conclusion that the depth of nevus infiltration is not only related to the size of nevus, but also related to the mutation of BRAF V600E gene in CMN. Additionally, the findings of this study expand the knowledge of genotype-phenotype correlation in CMN.

The monoclonal antibody of Ki-67 has been widely used in histopathology and shown to be of particular value for tumor grading and to correlate with patients’ survival in many human malignancies.^23^ But data on the expression of proliferative activity marker Ki67 in CMN are limited. Soyer included benign melanocytic nevi in his study and found significant differences in Ki-67 antigen expression between nevi, primary and metastatic malignant melanoma.^24^

BRAF V600E is observed in nevi with a high frequency^10^ and is a driver mutation in melanoma pathogenesis.^25^ BRAF V600E transiently activates nevi melanocytes proliferation and induces p16 that in response halts proliferating melanocytes.^26^ But the arrest is reversible since some nevi ultimately transform into skin melanomas.^27^ There is a dynamic balance between hyperplasia and inhibition of nevi, and if this balance is broken, it is likely to develop into malignant melanoma.

In the present study, the authors find that there is a statistically significant difference in proliferation activity between nevi with BRAF V600E mutation and the control group. Hence, with the BRAF V600E mutation, CMN can have more proliferative activity and maybe more susceptible to other irritant factors, such as sun exposure and friction, and would eventually progress to malignant melanoma.

As far as the authors know, we have demonstrated that the proliferation activity of CMN is positively correlated with the number of nevus cell nests but not with the nest size. Hence, the nest number may be an index of proliferative activity and could be used in a future study.

The above research is preliminary, and the results will have to be validated in a larger scale of study. Additional studies of nevi promise to inform understanding of the mechanisms of nevogenesis and the transition from benign neoplasm to malignancy.

## Conclusion

Our study demonstrates that BRAF V600E gene mutation can enhance proliferative activity and can affect the histopathological features of CMN in children. While on the other hand, the histopathological features of a CMN can provide relevant information on its molecular background, including the presence of BRAF V600E gene mutation, which is the most common driver of melanocytic tumors and the most common therapeutically targetable alteration in melanoma. A greater understanding of the genotype-phenotype correlation in CMN may facilitate a more accurate diagnosis and more proper management of these exceedingly common human melanocytic tumors.

## Financial support

This work was supported by the Research Foundation of the Capital Institute of Pediatrics(project number: LCPY-2021-04).

## Authors' contributions

Jianyou Chen: Approval of the final version of the manuscript; design and planning of the study; drafting and editing of the manuscript; collection, analysis, and interpretation of data; effective participation in research orientation; critical review of the literature; critical review of the manuscript.

Gaolei Zhang: Approval of the final version of the manuscript; design and planning of the study; drafting and editing of the manuscript; critical review of the literature.

Xiaoyan Liu: Approval of the final version of the manuscript; collection, analysis, and interpretation of data; effective participation in research orientation.

Ping Tu: Statistical analysis; approval of the final version of the manuscript; collection, analysis, and interpretation of data; effective participation in research orientation; critical review of the literature; critical review of the manuscript.

## Conflicts of interest

None declared.
